# Do Post Discharge Phone Calls Improve Care Transitions? A Cluster-Randomized Trial

**DOI:** 10.1371/journal.pone.0112230

**Published:** 2014-11-11

**Authors:** Christine Soong, Bochra Kurabi, David Wells, Lesley Caines, Matthew W. Morgan, Rebecca Ramsden, Chaim M. Bell

**Affiliations:** 1 Division of General Internal Medicine, University of Toronto, Toronto, Canada; 2 Department of Medicine, Mount Sinai Hospital, Toronto, Canada; Supportive care, Early DIagnosis and Advanced disease (SEDA) research group, United Kingdom

## Abstract

**Importance:**

The transition from hospital to home can expose patients to adverse events during the post discharge period. Post discharge care including phone calls may provide support for patients returning home but the impact on care transitions is unknown.

**Objective:**

To examine the effect of a 72-hour post discharge phone call on the patient's transition of care experience.

**Design:**

Cluster-randomized control trial.

**Setting:**

Urban, academic medical center.

**Participants:**

General medical patients age 18 and older discharged home after hospitalization.

**Main Outcomes and Measures:**

Primary outcome measure was the Care Transition Measure (CTM-3) score, a validated measure of the quality of care transitions. Secondary measures included self-reported adherence to medication and follow up plans, and 30-day composite of emergency department (ED) visits and hospital readmission.

**Results:**

328 patients were included in the study over an 6-month period. 114 (69%) received a post discharge phone call, and 214 of all patients in the study completed the follow outcome survey (65% response rate). A small difference in CTM-3 scores was observed between the intervention and control groups (1.87 points, 95% CI 0.47–3.27, p = 0.01). Self-reported adherence to treatment plans, ED visits, and emergency readmission rates were similar between the two groups (odds ratio 0.57, 95% CI 0.13–2.45, 1.20, 95% CI 0.61–2.37, and 1.18, 95% CI 0.53–2.61, respectively).

**Conclusions and Relevance:**

A single post discharge phone call had a small impact on the quality of care transitions and no effect on hospital utilization. Higher intensity post discharge support may be required to improve the patient experience upon returning home.

**Trial Registration:**

ClinicalTrials.gov NCT01580774

## Introduction

The transition from hospital to home can expose patients to adverse events during the post-discharge period [1], [2]. Preventable harm may arise from medication errors [1], discontinuity in care, inadequate communication between inpatient and outpatient providers [3], or lack of follow-up of pending inpatient results that may impact management [2], [4], [5]. In addition, patients and primary care providers (PCPs) report negative experiences following discharge from hospital. Indeed, patients often experience a “voltage drop” in support once leaving the hospital, resulting in feeling unprepared for discharge, and have challenges transitioning to normal life and translating knowledge into safe, health-promoting actions at home [6], [7]. Contributing factors include inadequate patient and caregiver education prior to discharge, lack of in-home resources, and access to treating physicians.

A follow-up telephone call is a simple method to connect with patients following discharge to review and reinforce discharge plans. However, previous studies examining phone calls to patients returning home were frequently conducted by a pharmacist and focused on medication management. A Cochrane systematic review found a high degree of heterogeneity and low methodological quality across studies, hence limiting a meaningful conclusion [8]. There is inconsistent evidence that post discharge phone calls, alone or as part of a discharge bundle, can affect readmission rates [9]. However, the effect of post discharge phone calls on the patient experience and on perceived comprehension of discharge instructions remains unclear.

Our institution publically reports a measure of the patient's experience on continuity and transition through a validated standardized commercial post discharge survey instrument (NRCC-Picker) [10]. Consistently, reported scores have been below benchmark and those of peer comparator organizations. Root cause analyses indicated inadequate patient and caregiver engagement and education, and lack of post discharge follow up as contributors. To address these issues, a cluster-randomized control study was conducted on general medicine wards to determine the impact of post discharge phone calls on the patient experience, as well as reinforcing patient discharge instructions.

## Methods

The protocol for this trial and supporting CONSORT checklist are available as supporting information; see Checklist S1 and Protocol S1.

### Study design

This is a cluster-randomized, single center study conducted in Toronto, Canada. Recruitment occurred between September 2012 and March 2013 and follow up ended July 2013. The unit of randomization was the treating medical team. Patients from two teams were randomized to receive a post discharge phone call from a Patient Navigator (PN). One Patient Navigator was assigned to each team, working Monday through Friday and maintained continuity with patients. Patients from the other two teams received usual care (no post discharge phone call). All patients were followed 30 days after discharge and contacted by a research assistant to collect survey outcome data. Patients and research staff were not blinded to intervention allocation.

### Participants

**Figure 1:**
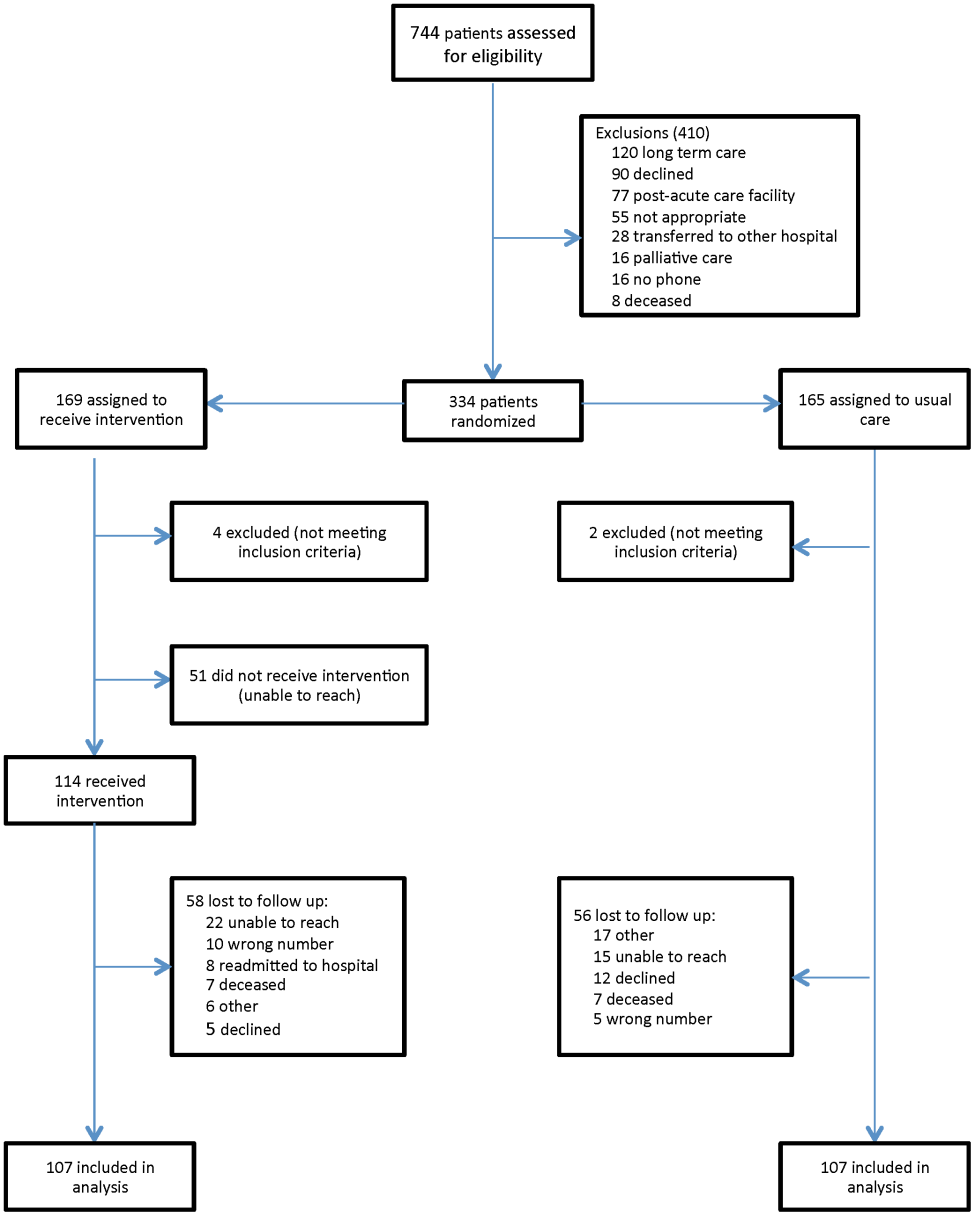
Patient enrolment and outcomes.

All patients aged 18 and older and discharged home from GIM inpatient wards were eligible for inclusion. Consecutive patients or their substitute decision maker were approached to participate in the study if they met inclusion criteria. Patients discharged to other facilities (e.g., long-term care, rehabilitation, other acute care facilities), those who died, or those without access to a phone were excluded. Patients were also excluded if the situation was not appropriate for a post discharge phone call (Figure 1). Examples include patients with cognitive improvement without available or willing caregivers to participate in survey measurement. Enrollment only occurred during regular working hours (weekdays 08:00–17:00, excluding holidays) due to limited availability of on-site research personnel.

### Setting

The study took place at Mount Sinai Hospital, a 446-bed academic medical centre in Toronto, Canada from September 2012 to March 2013. The general internal medicine (GIM) inpatient wards have 84 beds and had 3048 discharges in 2011. On GIM, four medical teams provide care for all inpatients. Each team comprises an attending physician, residents (one senior and 2 to 3 junior residents), medical students, a pharmacist, social worker and physical and occupational therapists. Patient navigators are non-clinician members of the team who facilitate inpatient scheduling of investigations and appointments and act as a resource to the medical team, the patient and their families. Engaged stakeholders supporting this initiative included administrative leaders, unit managers, and frontline staff, such as PNs.

### Intervention

The discharge process involves each patient receiving a copy of the electronic discharge summary and patient-specific instructions. In addition, the provider must review written discharge instructions with the patient and/or caregiver. Recent audits on the GIM inpatient units indicated that this occurs at rates close to 100%. The intervention began as a pilot of post discharge phone calls on one of the GIM wards by a nurse practitioner (RR), then expanded to involve a member of the medical team (i.e., the patient navigators). This ensured continuity as well as provided best knowledge of details specific to each patient.

The PN called a patient or caregiver within 3 days following discharge from hospital. Attempts were made during different hours of the day and on different days of the week. A minimum of 5 attempts was conducted. If the PN was unable to reach the patient, a voice message was left whenever possible. A standardized intervention phone script (Appendix S1) was designed to solicit information on general health status post discharge, comprehension of discharge instructions, and to reinforce instructions provided. The phone script was pilot tested among interprofessional team members and changes incorporated to increase reliability. The caller utilized a modified teach-back method [11] to educate the patient on discharge instructions, medications and follow up recommendations. If a clinical concern arose during the phone call, the PN relayed the information to the patient's inpatient care team for attention.

### Data collection

Experienced clinical research personnel reviewed the medical records of consenting patients and abstracted relevant data including demographics, main admission diagnosis, length of stay, and presence of PCP. Within 30 days of discharge, research personnel contacted consenting patients to conduct a brief interview including the Care Transitions Measure questionnaire (CTM-3) and information regarding follow up care and hospital utilization.

### Outcomes

The primary outcome measure was the Care Transition Measure score (CTM-3), a validated measure reflecting the quality of a patient's care transition that is associated with hospital utilization [12], [13]. We chose the CTM-3 score as the primary outcome measure because of its demonstrated validity and use in other care transition studies would allow for comparison. Secondary outcome measures included patient's self-reported rates of adherence to medications and post discharge plans (including follow up appointments), and unplanned hospital utilization (30-day emergency department visit or readmission to any hospital in the local health region as verified per patient self-report and/or available electronic medical records). Intervention fidelity was captured through rates of completed post-discharge phone calls and assessment of PN's adherence to the script. This occurred through direct observation by the principal investigator (CS). Other measures included the number of medical issues requiring physician or clinician response, and the number of attempts and time required to complete phone calls.

We administered a telephone survey 30 days following discharge from hospital to all patients. Patients were asked whether they understood the medications and follow up instructions. The responses generated a CTM-3 score ranging from 0 to 100 as an objective measure of discharge instruction comprehension, with higher scores indicating higher quality care transition. Self-reported adherence to medication and follow up instructions and appointments were also solicited and cross-referenced with documented instructions in discharge summaries. In addition, any reported unplanned visits to the emergency department (ED) or readmission to any hospital was confirmed through electronic health record review, including that of regional hospitals participating in a shared electronic patient information system.

### Sample size

A previous study using the CTM-3 in a similar patient population found a mean of 80.3 with a standard deviation of 19.6 [14]. Although there is little guidance as to a minimum clinically significant change in CTM-3 score, the author and proprietor of this measure suggests a minimum change of 5–7 points to be meaningful to administrators [15]. With an alpha-error of 0.05 (2-sided) and a beta-error of 0.20 (1-sided), the unadjusted total sample needed to detect a 7-point difference in CTM-3 scores was 95 in each arm (based on 85% power and an estimated intra-class correlation coefficient of <0.05). Assuming a 50% response rate of patients completing the outcome survey, we calculated an adjusted sample size of 92 in each arm, or 184 patients in total, adjusting for clustering effects. Given that over an average month, 200 patients are discharged from the GIM service at our hospital and assuming a conservative historical trend of 70% of patients returning home and a combined 20% eligibility, participation and enrollment rate, we elected to conduct the study over a 6-month period.

### Statistical analysis

Descriptive analyses were used to summarize preliminary results. Continuous variables were compared using the Mann-Whitney test and categorical variables with Fisher's exact test or Chi-square. Statistical comparison between intervention and control groups was performed using a generalized estimating equation (GEE) approach, adjusting for clustering at the provider and unit levels [16]. Given the small number of clusters a post-hoc sensitivity analysis using a mixed-effect model was also conducted. Missing data cannot be assumed to be at random and thus not amenable to the usual imputation strategies.

### Ethics

The Mount Sinai Hospital Review Ethics Board approved the study protocol. All participants provided written informed consent. The trial was registered with ClinicalTrials.gov (NCT01580774).

## Results

Eligible patients were recruited between September 2012 and March 2013, from the GIM inpatient wards. (Figure 1) Recruitment ended when the sample size was reached. The mean age of participants was 64 years. About 60% were women, most had family physicians, and were in hospital for just under 8 days. The most common diagnosis was pneumonia. There were no differences between the two groups in terms of baseline demographic characteristics (Table 1).

**Table 1:** Characteristics of patients.

	Intervention (n = 165)	Control (n = 163)
Mean age at discharge, year (SD)	65 (21)	63 (20)
Female, no (%)	102 (62)	98 (60)
Most responsible diagnosis, n (%)		
Pneumonia	7 (4)	11 (7)
Fever/sepsis	6 (4)	5 (3)
Gastrointestinal bleed	6 (4)	4 (2)
Urinary tract infection or pyelonephritis	4 (2)	7 (4)
Dementia, delirium or confusion	7 (4)	2 (1)
COPD/asthma	5 (3)	3 (2)
Stroke	5 (3)	8 (5)
Heart failure	4 (2)	3 (2)
Cellulitis	5 (3)	5 (3)
Other	116 (70)	115 (71)
Mean length of stay, days (SD)	7.6 (6.0)	8.0 (7.0)
Presence of family physician, n (%)	144 (87)	139 (85)
Admission to geriatric unit, n (%)	121 (73)	134 (82)

p>0.05 for all comparisons.

Fidelity of the intervention was 69% (114 out of 165). The PNs attempted to improve fidelity by increasing the frequency of attempts (beyond 5) to contact patients but calls were often not returned. The majority of patients who responded to the intervention phone call were reached with less than 5 attempts. Reasons for not reaching patients in the intervention arm included loss to follow up (27), readmission to hospital (9), wrong phone number (5), deceased (2), declined (2), and other (6). Readmission to hospital accounted for 9 (18%) patients in the intervention arm who could not be reached for a post discharge phone call. The 30-day survey response rate was 65% (214 out of 328) (Table 2). CTM-3 scores for the intervention group was 73.1±19.5 and 71.3±18.8 for the control group. An intention-to-treat analysis revealed a small statistically significant difference in CTM-3 scores between the two groups (1.87 points, 95% CI 0.47–3.27, p = 0.01). On-treatment analysis and adjusting for geriatric unit assignment did not change the results.

**Table 2:** Patient Outcomes.

	Intervention	Control	P
CTM-3 score, mean (SD)	73.1 (19.5)	71.3 (18.8)	0.01
Adherence to post discharge follow up appointments, n (%)	90 (84)	96 (90)	0.22
Medication adherence, n (%)	91(85)	95 (89)	0.42
ED visit, n (%)	22 (21)	19 (18)	0.60
Readmission, n (%)	15 (14)	13 (12)	0.68

Secondary outcomes of self-reported adherence to treatment plans and medications, and 30-day ED visit and readmission were similar between the two groups (odds ratio 0.57, 95% CI 0.13–2.45, 1.20, 95% CI 0.61–2.37, and 1.18, 95% CI 0.53–2.61, respectively) (Table 2).

### Sensitivity analysis

Additional post-hoc sensitivity analysis using a mixed-effect model revealed the absence of clustering effect (i.e., intra-class correlation coefficient of zero) indicating a standard analysis was acceptable. The lack of a cluster effect was evident in the primary outcome as well as the secondary outcomes. Therefore the primary outcome was analyzed with linear regression and secondary outcomes were analyzed with logistic regression. The sensitivity analysis revealed similar estimate of difference but the effect of the primary outcome was no longer statistically significant (2.64, 95% CI -2.51–7.78, P = 0.31).

## Discussion

We conducted a cluster randomized control trial comparing post discharge phone calls with usual care and found minimal improvement in CTM-3 scores in the intervention group over the control. However, the effect was small and may lack clinical relevance to clinicians and administrators. To our knowledge, this is the first study evaluating the effect of post discharge phone calls on a validated measure of the quality of care transitions, the CTM-3 score.

The association between suboptimal transitions of care and readmission rates [1] has stimulated great interest in post discharge efforts, such as phone calls, to support patients returning home. The results of our study are consistent with previous efforts that failed to demonstrate a meaningful effect of post discharge phone calls on readmission. However, several studies reported improved patient satisfaction scores. Dudas and colleagues conducted a randomized control trial of a pharmacist-led post discharge phone call intervention [17]. Outcomes measured were patient satisfaction scores, 30-day ED visits, and readmission. Implementation fidelity was similar to our study, with higher reported satisfaction with medication instruction and lower ED visit rates in the intervention arm. However, the study was limited by a small sample size and possible clustering effects. A second study used two phone calls to patients discharged from medicine one week and one month post discharge [18]. Although findings shared similar limitations, the authors found no differences in the primary end-point of readmission. A Cochrane systematic review examining the effects of follow up phone calls on patient outcomes found some evidence of improved effects such as adherence to post discharge appointments without evidence of adverse events [8]. However, heterogeneity, low methodology, and a risk of bias precluded a firm conclusion on the effects of discharge phone calls. In the majority of these studies, a hospital-based individual conducted the phone calls. It is unclear whether this contributed to the negative results and whether calls from outpatient-based primary care teams would be more effective. Finally, our intervention differed from previous studies in that a non-clinician conducted the phone calls, possibly reducing impact. A previous negative telephone study also involved a non-clinician caller [19].

Post discharge phone calls are but one component of “discharge bundles” that aim to prepare a patient for discharge. Over-reliance on this intervention to impact on the complex phenomenon of readmission may not be successful [20]. Our study showed that a single post discharge phone call had little impact on an objective measure of care transition. This observation supports the concept that care transition interventions may be most effective when delivered in a bundle where the effect on outcomes can be additive [9]. Other single intervention studies showed minimal impact on CTM-3 scores with absolute differences similar to our study [14], [21] whereas a study involving a multi-site, multi-faceted interventions resulted in greater improvement [22]. Although our intervention was simple and low cost (phone calls were incorporated into daily workflow), ideal post discharge care may require more intensive interventions such as repeated phone calls by a clinician or home visits as described in Project RED [23] and BOOST [24] to have a more appreciable effect. Finally, high resource-intensive interventions may be best targeted toward select patients with complicated discharge plans at risk of adverse events [20]. The decision to incorporate post discharge phone calls will require careful consideration of several factors. Patient factors such as complexity of disease and care plans, and poor social supports may warrant post discharge phone calls. How these calls fit into provider workflow, other discharge bundle interventions, and associated personnel costs need to be weighed against limited efficacy. Our next steps include evaluating discharge phone calls in select high-risk patients and in concert with other post discharge interventions.

Our study has several limitations. First, the implementation fidelity of the discharge phone calls was 69%, possibly reducing the effect of the intervention. This is however, consistent with other studies of post discharge phone calls [8] and an on-treatment analysis yielded similar results. Second, the primary outcome of CTM-3 scores was collected 30 days post discharge and may be subject to recall bias. Measurement of outcomes one month following the intervention was important to determine any long-term effects and sustainability of the phone calls on the patient experience. Given that readmission to hospital can occur throughout the 30-day period following discharge, it was important to capture the impact of the intervention during this time frame [25]. In addition, CTM-3 scores are frequently measured 30-days post discharge [8]. Third, we only measured self-reported adherence to treatment plans and ED visits and readmission rates. In addition, the study was not powered to detect secondary outcomes. However, we were able to confirm hospital utilization within our local health region through available electronic health records. Fourth, we enrolled all eligible patients rather than targeting those who may benefit most and/or require intensive support following discharge, possibly diluting the effects of the intervention. We plan to study our intervention in patients discharged with complex care plans at high risk of adverse events. Fifth, the provider teams and survey assessors were unblinded, potentially introducing bias. However, frontline staff frequently rotated to other facilities and was often unaware of the intervention. Sixth, patients lost to follow up represented the majority of missing data which cannot be assumed to be at random. This precluded imputation strategies to address missing data and may have introduced bias. Last, this study was conducted in a single academic medical centre and results may not be generalizable to other settings although our patient population and demographics are typical of many acute care facilities.

A single post discharge phone call for patients returning home from hospital failed to produce a meaningful impact on the patient's discharge experience. Our observations support a focus on higher intensity support in the post discharge period in select patients to aid transition to normal home life.

## Supporting Information

Checklist S1 **CONSORT checklist of information.**
(DOC)

Protocol S1 **Post discharge phone call study protocol.**
(DOCX)

Appendix S1 **Post discharge intervention phone call script.**
(DOCX)
