# Supplementary material for: Do Post Discharge Phone Calls Improve Care Transitions? A Cluster-Randomized Trial
Source: PLoS One. 2014 Nov 11;9(11):e112230. doi: 10.1371/journal.pone.0112230 (PMC4227814; doi:10.1371/journal.pone.0112230)
Supplement: Protocol S1 — Post discharge phone call study protocol. (DOCX) [file pone.0112230.s002.docx]

**Post discharge follow-up phone calls Protocol**

**Clinical Trials Protocol: MSHCANADA-CSCB**

**Introduction**

Currently, discharge from hospital in many institutions is a confusing process for patients filled with uncertainty and potential for harm. For instance, 1 in 5 discharges results in a post discharge adverse event, many of which are related to medication errors.^1,2^ These may lead to serious harm and possibly require readmission to hospital. Prior research suggests conducting post discharge follow up phone calls may improve patient satisfaction, reduce unnecessary hospital visits, and improve adherence to medications and discharge care plans.^3^ However, there are few studies focused on general medicine patients and most were of small sample size of low methodological quality. We propose a randomized controlled trial to determine the effect of post discharge phone calls on patient outcomes in general medical patients.

**Hypothesis**

A post discharge follow up phone call to general medical patients discharged home will improve Care Transition Measure scores, adherence to treatment plan (including medication) and patient satisfaction, and decrease medication error and subsequent hospital utilization including readmission and emergency department visits in the Local Health Integration Network.

**Significance**

The transition from hospital to home is a critical period during which significant harm or avoidable resource utilization can result from lack of supports and poor understanding of discharge plans. Telephone follow-up calls after discharge has been studied in small single-center trials and as a part of a coordinated, multi-layered discharge process but its direct effectiveness is not known. Understanding the impact of this simple intervention on patient outcomes is an important step towards improving patients’ discharge from hospital.

**Methods**

Setting

Mount Sinai Hospital, an academic medical centre with general medicine inpatient units.

Patients

All patients admitted to a general internal medicine ward who survive to hospital discharge home.

Exclusion criteria: Patients who do not have a telephone, patients discharged to a facility (rehabilitation, nursing, other acute care facility), patients who decline to participate.

Study design:

Cluster randomized control trial in a teaching hospital. Clinical teaching unit (CTU) “teams” of patients will be randomized to receive either routine care (no telephone follow up) or intervention (telephone follow up within 72 hrs of discharge). All patients will be followed 30 days after discharge and contacted to collect survey outcome data. Clinical outcomes data will be collected from hospital database.

Survey Design:

Intervention group will receive a phone call within 72 hours after discharge addressing the following domains:

- Patient status since discharge
- Patient’s understanding of discharge instructions
- Patient’s understanding of changes in his/her medications
- Any follow up visits to family practitioner or other specialists
- Any concerns or questions outstanding

All patients will be contacted at 30-days post-discharge to complete a survey regarding outcomes. The survey will contain the Care Transitions Measure-3 (CTM-3), a validated tool to evaluate patients’ perception on coordination of hospital discharge care in addition to questions pertaining to hospital utilization, and rates of adherence to treatment plans and follow up. Follow up surveys may be conducted up to 90 days post discharge.

Callers will follow a standardized script. Patients will be contacted at least five times (unless respondent refuses to participate) to complete the intervention and follow-up survey. Attempts must be made on different days and at different times of the day.

Data Collection

| **Variable** | **Measurement** | **Variable Type** |
| --- | --- | --- |
| *Patient Demographics*  Age  Sex  Admitting diagnosis  Most responsible diagnosis  Family physician  Mean length of stay | Hospital Chart | Continuous  Binary  Categorical  Categorical  Binary  Continuous |

Primary Outcome

Change of 5-points on the Care Transition Measure-3 score (survey)

Secondary Outcomes

30-day Hospital Readmission (TC-LHIN data)

ED visits within 30 days or weeks (TC-LHIN data)

Patient satisfaction (survey)

Adherence to treatment plan (survey)

Rates of follow up with PCP/outpatient providers (survey)

**Statistical Analysis**

Sample size justification

A previous study using the CTM-3 in a similar patient population found a mean of 80.3% with a standard deviation of 19.6.^4^ We determined that a 7-point absolute difference in CTM-3 scores would be clinically relevant to physicians and hospital administrators. With an alpha-error of 0.05 (2-sided) and a beta-error of 0.20 (1-sided), the unadjusted total sample needed to detect a 7-point difference in CTM-3 scores was 95 in each arm. Assuming a 50% response rate of patients completing the post-discharge survey, we calculated an adjusted sample size of 190 in each arm, or 380 patients in total. Given that over an average month, 254 patients are discharged from the GIM service at our hospital and assuming a conservative historical trend of 80% of patients returning home and a 50% participation rate, we elected to conduct the study over a 4-month period.

Descriptive analyses will be used to summarize results (e.g., means, medians, and standard deviations). Continuous variables will be compared using Mann-Whitney test and categorical variables with Fisher’s exact test or Chi-square.

**Budget Justification**

As the proposed study is part of a quality improvement initiative, members of current medical care teams will be required to perform the post-discharge phone calls as a matter of routine care. Funding application has been put forth to the Office of Patient Experience and Outcomes at Mount Sinai Hospital to facilitate the cost of a research coordinator (0.5 FTE) to track the patients enrolled and to conduct follow-up telephone surveys.

**References**

^1^Forster AJ, et al. The incidence and severity of adverse events affecting patients after discharge from the hospital. Ann Int Med. 2003;138:161-7.

^2^Forster AJ, et al. Adverse events among medical patients after discharge from hospital. CMAJ. 2004;170:345-9.

^3^Mistiaen P. Telephone follow-up, initiated by a hospital-based health professional, for postdischarge problems in patients discharged from hospital to home. Cochrane Database of Systematic Reviews 2006;(4):CD004510

^4^Maslove, D. M., Leiter, R. E., Griesman, J., Arnott, C., Mourad, O., Chow, C.-M., & Bell, C. M. (2009). Electronic Versus Dictated Hospital Discharge Summaries: a Randomized Controlled Trial. *JGIM*; *24*(9), 995–1001.
