# Supplementary material for: Do Post Discharge Phone Calls Improve Care Transitions? A Cluster-Randomized Trial
Source: PLoS One. 2014 Nov 11;9(11):e112230. doi: 10.1371/journal.pone.0112230 (PMC4227814; doi:10.1371/journal.pone.0112230)
Supplement: Appendix S1 — Post discharge intervention phone call script. (DOCX) [file pone.0112230.s003.docx]

**Post discharge phone call study**

**Intervention phone call – Algorithm**

Hello, this is (insert caller name) calling from Mount Sinai Hospital. I am part of the medical team that provided care for you during your recent hospitalization (insert dates ). I am calling to see how you have been doing since your discharge. Your participation is voluntary and will not affect your health benefit.

Question #1

Question #2 (compare responses to dc summary)

1 = completely inaccurate, 2 = somewhat inaccurate, 3 = 50-50 accurate/inaccurate, 4 = somewhat accurate, 5 = completely accurate.

***Based on dc summary, caller will now provide patient with correct information and reinforce importance of medication adherence and need to follow up with physicians/scheduled tests. Caller will utilize teach back to ensure patient understanding. Caller will assist in appointment scheduling if patient has not done so. Caller may provide home care contact number for relevant issues, if necessary.

Question #3

“Do you have any concerns or questions?”

- record and pass on to physician if patient requests.
